# Risk Assessment of Insecticides Used in Tomato to Control Whitefly on the Predator *Macrolophus basicornis* (Hemiptera: Miridae)

**DOI:** 10.3390/insects12121092

**Published:** 2021-12-07

**Authors:** Thaís Fagundes Matioli, Mariana Rosa da Silva, Juliano de Bastos Pazini, Geovanny Barroso, Júlia Gabriela Aleixo Vieira, Pedro Takao Yamamoto

**Affiliations:** Department of Entomology and Acarology, “Luiz de Queiroz” College of Agriculture, University of São Paulo (ESALQ/USP), Piracicaba 13418-900, Brazil; marianarosa.silva@usp.br (M.R.d.S.); julianopazini@usp.br (J.d.B.P.); geovannybarroso@usp.br (G.B.); julia.aleixo@usp.br (J.G.A.V.); pedro.yamamoto@usp.br (P.T.Y.)

**Keywords:** biological control, ecotoxicology, integrated pest management, natural enemy

## Abstract

**Simple Summary:**

The whitefly *Bemisia tabaci* is a problem in tomato crops worldwide. The use of chemicals is one method to control this pest. Predators from the family Miridae have been used in Europe as biological control agents. We tested the insecticides most often used to control *B. tabaci* in tomato fields in Brazil for compatibility with the native Brazilian mirid *Macrolophus basicornis*. The results showed that regarding lethality, buprofezin, cyantraniliprole and spiromesifen were reduced-risk insecticides. Acetamiprid, bifenthrin, etofenprox + acetamiprid and pyriproxyfen + acetamiprid were considered broad-spectrum insecticides. The insecticides were also tested to be classified ecologically and were found to be safe, except for acetamiprid that was moderately toxic. Overall, our findings indicated that it is possible to use *M. basicornis* as a biological agent to control *B. tabaci* in tomato crops by means of pest management strategies that are compatible with agrochemicals in current use.

**Abstract:**

The generalist mirid predator *Macrolophus basicornis* may contribute to Integrated Pest Management (IPM) of *Bemisia tabaci* in tomato crops. It is important to know the compatibility of the chemicals used to control this pest with this promising biological control agent. Seven insecticides were tested to investigate their toxicity to the predator. For four of the products, the LC_50_ for adults were determined. Buprofezin, cyantraniliprole and spiromesifen did not cause lethality and were classified as harmless. Acetamiprid, bifenthrin, etofenprox + acetamiprid and pyriproxyfen + acetamiprid caused acute toxicity and were classified as harmful. LT_50_ for all harmful insecticides were relatively low, ranging from 1.8 to 3.2 days. Moreover, these four insecticides have low LC_50_, with acetamiprid (0.26 mg a.i. L^−1^) as the lowest, followed by bifenthrin (0.38 mg a.i. L^−1^), etofenprox + acetamiprid (4.80 mg a.i. L^−1^) and pyriproxyfen + acetamiprid (8.71 mg a.i. L^−1^). However, the calculated risk quotient (RQ) values demonstrated that these insecticides were mostly ecologically safe for this predator, except for acetamiprid, classified as slightly to moderately toxic. The present study can contribute to the use of *M. basicornis* as a biological control agent on tomato crops and to compatible use with the insecticides tested, according to IPM strategies.

## 1. Introduction

The family Miridae contains a significant number of predator species used in augmentative biological control in tomato crops [1,2]. The genus *Macrolophus* has been used in Europe to control *Tuta absoluta* (Meyrick) (Lepidoptera: Gelechiidae) and *Bemisia tabaci* (Genn.) Biotype B (Hemiptera: Aleyrodidae), the main tomato pests [2]. In Brazil, *Macrolophus basicornis* (Stal) (Hemiptera: Miridae) has a considerable potential to be reared in biofactories and released in the field to control the whitefly *B. tabaci* [3,4,5,6,7,8,9]. Some studies have shown that this natural enemy can easily establish in the field because of its zoophytophagy, a trait that aids it to remain where it is released without the presence of prey, since it can consume the sap from the crop [7,10,11,12]. Despite the benefits of using this natural enemy, *B. tabaci* is controlled by different insecticides, due to its direct and indirect damage on tomato [13,14], which may harm this possible new biological control agent. 

It is important to understand the acute toxicity of insecticides used in pest control and the ecological risks to natural enemies prior to introducing a new biological control agent in any crop [15,16]. Hence, the development of new strategies in integrated pest management (IPM) programs involves compatibility studies of tactics, especially chemical and biological control [17]. Recent studies have demonstrated the effects of chemical products used to control *T. absoluta* on the predator *M. basicornis* [18,19,20,21]. Studies are needed to assess the toxic effects on *M. basicornis* using chemical products against *B. tabaci*.

Both the whitefly and the mirid predator have sucking mouthparts that introduce the stylets into the tissues [4,14]. Many insecticides used to control *B. tabaci* are systemic and can affect the mirid, which feeds on plant tissue as a source of water and nutrients. The insecticides function by contact exposure to reach the pest nymphs and adults that remain on the leaves and residues may harm *M. basicornis* individuals on treated surfaces. Chemical groups have different modes of action on pests. Many are broad-spectrum and can kill a wide range of many natural enemies [22,23], including *M. basicornis*. Other, reduced-risk insecticides are more selective to the predators and cause no or low lethal effect [18,20,24].

The first step of risk assessment is to determine the acute toxicity of commonly used insecticides to natural enemies in a laboratory bioassay, using the recommended field rates. One way to classify the chemicals is according to the International Organization for Biological and Integrated Control of Noxious Animals and Plants (IOBC), evaluating their lethal effect on the target species to determine the physiological selectivity [25]. Another important classification is the risk quotient (RQ), which classifies the chemicals that will be ecologically selective, related to the ways that the insects are exposed in the field [26,27]. This evaluation can help to determine the possible risks of pesticides to natural enemies in the field, quantifying the concentrations for parasitoids and predators, as estimated for *Encarsia formosa* Gahan (Hymenoptera: Aphelinidae) [28], three *Trichogramma* species (Hymenoptera: Trichogrammatidae) [27,29] and the mirid *Cyrtorhinus lividipennis* Reuter (Hemiptera: Miridae) [26,30]. 

The present study was conducted to assess the acute toxicity and RQ of seven insecticides commonly used to control *B. tabaci* in tomato crops on the mirid predator *M. basicornis*. We investigated the acute toxicity and median lethal concentration (LC_50_) of the insecticides that are currently most often used (acetamiprid, bifenthrin, buprofezin, cyantraniliprole, etofenprox + acetamiprid, pyriproxyfen + acetamiprid and spiromesifen). We hypothesized that it is possible to find compatible insecticides with the predator to enable it to be used as a biological control agent, adding to the IPM tactics.

## 2. Materials and Methods

### 2.1. Insects

Individuals of *M. basicornis* were obtained from the established rearing colony, more than eight generations old, at the Laboratory of Insect Biology in the Entomology and Acarology Department, “Luiz de Queiroz” College of Agriculture (ESALQ/USP), Piracicaba, Brazil. The insects were originally collected in the state of Minas Gerais, Brazil (21°08.596′ S and 45°03.466′ W, 808 m altitude) in tobacco (*Nicotiana tabacum* L.) fields. The method used was proposed by Bueno et al. [4], in which adults and nymphs were kept on tobacco plants in acrylic cages (60 × 30 × 30 cm) and fed with eggs of *Ephestia kuehniella* Zeller (Lepidoptera: Pyralidae) offered ad libitum. The cages were kept in a climate-controlled room at 25 ± 2 °C, 70 ± 10% RH and 12:12 h (L:D). Before the experiments, adults were kept in cages with tobacco plants for oviposition. After 48 h, the plants were moved to an insect-free cage. This made it possible to obtain predators with the same age, either third-instar nymphs or adults (<3 days old) from the plants [20].

### 2.2. Insecticides

The commercial insecticides are registered for the control of *B. tabaci* in Brazilian tomato crops and were tested on *M. basicornis* at the highest recommended field doses (Table 1). 

### 2.3. Insecticide Exposure for Testing Acute Toxicity

Five-week-old tomato plants (cv. Santa Clara) grown in greenhouse conditions were sprayed with each of the insecticides listed in Table 1, using a hand-held sprayer (Light Sprayer—Breeze, 500 mL capacity; Guarany; São Paulo, Brazil) until the run-off point (~50 mL per plant). Distilled water was used as the control treatment [31]. 

After drying for 2 h, the leaves were collected from treated plants and transferred to the laboratory. Each leaf had its petiole inserted into a flask (20 mL) filled with water to maintain turgidity during the bioassay and provided a lid with an opening for the petiole (Supplementary Material Figure S1). Each tube was transferred to a cage (12 cm high × 5 cm diameter) (PET crystal, 500 mL; Copozan, Otávio Dalazen, Brazil), with each unit representing one repetition. In each cage, 15 adults of *M. basicornis* (<3 days old) or 15 third-instar nymphs were released and the cage was covered with voile fabric to prevent accumulation of toxic gases and retain the insects. *M. basicornis* individuals were fasted for 24 h before the beginning of the experiments to ensure that they started to feed as soon as they came into contact with the insecticide residues. As an alternative food source for *M. basicornis*, *E. kuehniella* eggs (0.4 g) were offered per cage. The design was randomized with 6 replicates per treatment.

*M. basicornis* were left on the treated leaves for 72 h under controlled room conditions (25 ± 2 °C, 70 ± 10% RH and 12:12 h L: D). After this period, untreated leaves were provided to assess the survival rate and median lethal time (LT_50_). The insects’ survival was assessed every 24 h. The insects were considered dead when they were unable to walk at least the distance of their own body length after being touched with a fine brush.

### 2.4. Determination of LC_50_ of Harmful Insecticides

The median lethal concentration (LC_50_) was estimated for those insecticides that were harmful to adults of *M. basicornis* in the acute toxicity test (Section 2.3). The procedures for the LC_50_ bioassays were similar to the methods in Section 2.3. The design was completely randomized, with 6 replicates per treatment and 15 *M. basicornis* adults (<3 days old) in each cage. The bioassays were performed with different concentrations per insecticide, below the recommended field concentration (Table 1), according to procedures described by Finney [32]. The following insecticide concentrations (in mg a.i. L^–1^) were used: five concentrations of acetamiprid (0.03, 0.3, 1.0, 15 and 30); eight concentrations of bifenthrin (0.015, 0.075, 0.15, 0.75, 1.5, 7.5, 15 and 22.5); six concentrations of etofenprox + acetamiprid (0.18, 0.93, 9.34, 18.68 and 93.4); and five concentrations of pyriproxyfen + acetamiprid (0.09, 0.45, 0.9, 4.5, 9.0 and 45.0). Mortality was assessed 72 h after insecticides exposure to calculate the LC_50_ and the live insects were checked every 24 h. The insects were considered dead when they were unable to move at least the distance of their own body length after being touched with a fine brush.

### 2.5. Statistical Analysis

The data for the total number of live insects per replicate after 24, 48 and 72 h were checked for normality and homoscedasticity using the Shapiro–Wilk and Bartlett tests. If the assumptions of ANOVA were met, one-way ANOVA with Scott–Knott post-hoc (*p* < 0.05) was used to ascertain differences among treatment means. If the data did not satisfy the normality and variance homogeneity, Kruskal–Wallis non-parametric one-way ANOVA with Dunn with Bonferroni correction post-hoc (*p* < 0.05) was used through the “ExpDes”, “easyanova” and “dunn.test” packages in the R software [33]. The mortality percentage values after 72 h were corrected according to the Schneider–Orelli formula [34]: M_a_ (%) = [(M_t_ − M_c_)/(100 − M_c_)] × 100, where M_a_ is the corrected mortality, M_t_ the mortality observed in the treatment and M_c_ the control mortality. 

The data for survival of mirids exposed to insecticides over time were analyzed using Kaplan–Meier estimators (Log-Rank method). The survival curves and the median lethal time (LT_50_) were compared using the Holm–Sidak test, in SigmaPlot version 12.3 (Systat Software, San José, CA, USA). 

The data obtained from the tests to estimate the LC_50_ were submitted to a binomial model with the log-logistic regression (*drfit*) function for dose-response analysis in the statistical program R [33,35].

### 2.6. Toxicity Classification

Insecticides were classified in the toxicological categories of residual effects for tests in extended laboratory analysis, with the corrected mortality (M_a_) according to the IOBC, in which: class 1 = harmless (M_a_ < 25%); class 2 = slightly harmful (25 ≤ M_a_ ≤ 50%); class 3 = moderately harmful (51 ≤ M_a_ ≤ 75%); and class 4 = harmful (M_a_ > 75%) [25].

To assess the ecological risk of the harmful insecticides, the risk quotient (RQ) was calculated from the LC_50_ values at 72 h after exposure, based on the formula: RQ = recommended field rate (g a.i. ha^−1^)/LC_50_ of beneficial insects (mg a.i. L^−1^). To understand the ecological selectivity of the harmful insecticides, the calculated RQ values estimate the possible effect that can occur in the field. According to the results, the insecticides were classified as safe (RQ < 50), slightly to moderately toxic (50 < RQ ≤ 2500) or dangerously toxic (RQ > 2500) [26].

## 3. Results

The toxicity for nymphs and adults of the insecticides tested varied widely. The number of live insects in the groups exposed to buprofezin, cyantraniliprole and spiromesifen were similar to the control treatment, while the number of live insects exposed to acetamiprid, bifenthrin, etofenprox + acetamiprid and pyriproxyfen + acetamiprid significantly differed from the other treatments for both third-instar nymphs (Table 2) and adults (Table 3). The data obtained for the most lethal insecticides showed an increasing toxicity over time for nymphs (24 h: χ^2^ = 37.3, *df* = 7, *p* < 0.001; 48 h: χ^2^ = 36.7, *df* = 7, *p* < 0.001; 72 h: χ^2^ = 39.0, *df* = 7, *p* < 0.001) (Table 2) and for adults (24 h: F = 34.9, *df* = 7, *p* < 0.001; 48 h: F = 34.9, *df* = 7, *p* < 0.001; 72 h: F = 34.9, *df* = 7, *p* < 0.001) (Table 3). 

After 72 h of exposure of third-instar nymphs to insecticide residues on tomato leaves, buprofezin, cyantraniliprole and spiromesifen caused less than 1% mortality (Table 2). For acetamiprid, bifenthrin, etofenprox + acetamiprid and pyriproxyfen + acetamiprid, mortality ranged from 91.3% to 100% (Table 2). For adults, spiromesifen, buprofezin and cyantraniliprole caused 0, 2.1 and 3.5% mortality, respectively (Table 3). Pyriproxyfen + acetamiprid and bifenthrin reached 87.4 and 80.3% mortality, while etofenprox + acetamiprid and acetamiprid were the most harmful to adults, causing 96.5 and 98.6% mortality (Table 3).

According to IOBC classifications for acute toxicity, buprofezin, cyantraniliprole and spiromesifen are harmless (M_a_ < 25% = class 1) to nymphs and adults of *M. basicornis*. Acetamiprid, etofenprox + acetamiprid, pyriproxyfen + acetamiprid and bifenthrin are categorized as harmful (M_a_ > 75% = class 4) to this predator (Table 2 and Table 3).

Survival rates for nymphs and adults after 72 h exposure to insecticides showed significant differences among treatments (nymphs: χ^2^ = 686.96, *df* = 7, *p* < 0.001; adults: χ^2^ = 661.1, *df* = 7, *p* < 0.001). Buprofezin, cyantraniliprole and spiromesifen were similar to the control (Table 4).

**Figure 1:**
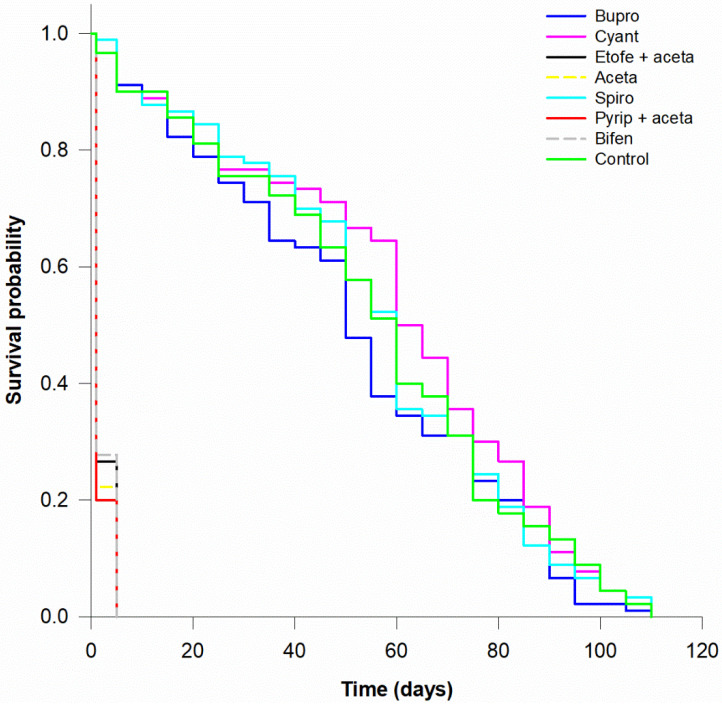
Survival curves for *Macrolophus basicornis* third-instar nymphs exposed to residues of buprofezin (Bupro), cyantraniliprole (Cyant), etofenprox + acetamiprid (Etofe + aceta), acetamiprid (Aceta), spiromesifen (Spiro), pyriproxyfen + acetamiprid (Pyrip + aceta), bifenthrin (Bifen) and control (water). The insects were in contact with the residues on tomato leaves for 72 h in controlled conditions.

**Figure 2:**
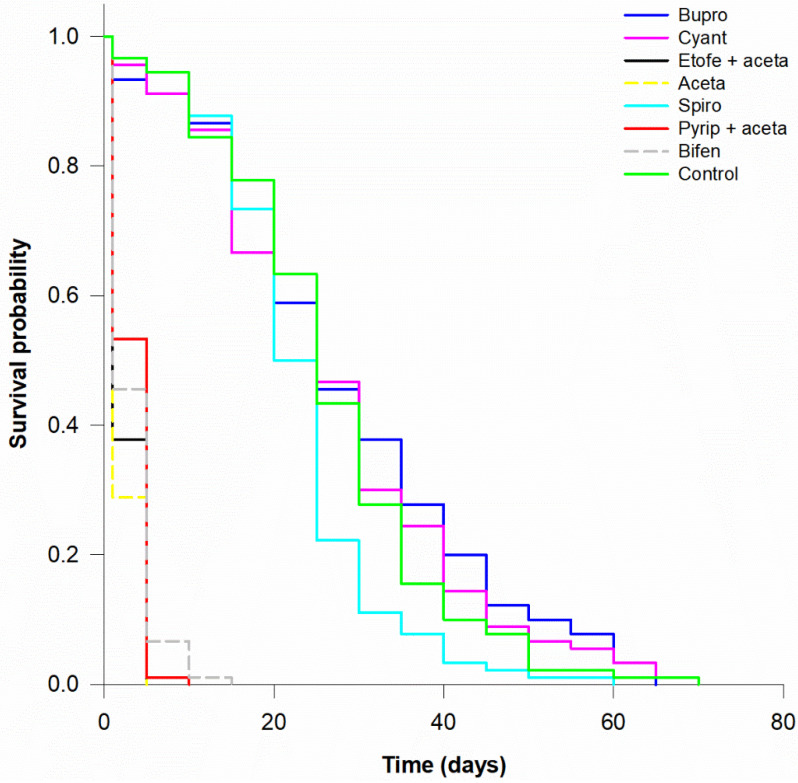
Survival curves for *Macrolophus basicornis* adults exposed to residues of buprofezin (Bupro), cyantraniliprole (Cyant), etofenprox + acetamiprid (Etofe + aceta), acetamiprid (Aceta), spiromesifen (Spiro), pyriproxyfen + acetamiprid (Pyrip + aceta), bifenthrin (Bifen) and control (water). The insects were in contact with the residues on tomato leaves for 72 h in controlled conditions.

In comparison to the control group, the LT_50_ values of acetamiprid, etofenprox + acetamiprid, pyriproxyfen + acetamiprid and bifenthrin were reduced by the acute toxicity of these active ingredients, ranging from 1.8 to 2.1 days for nymphs and 2.2 to 3.2 days for adults (Table 4). In the survival curves, nymphs (Figure 1) were more vulnerable to the harmful insecticides than adults (Figure 2).

The median lethal concentration (LC_50_) values are shown in Table 5 for acetamiprid, bifenthrin, etofenprox + acetamiprid and pyriproxyfen + acetamiprid, at 72 h after exposure of the adults to insecticide residues. Acetamiprid and bifenthrin had similar LC_50_ values and etofenprox + acetamiprid and pyriproxyfen + acetamiprid were less toxic and with similar LC_50_ values, with overlapping confidence intervals from 3.28 to 11.25 mg a.i. L^−1^ (Table 5).

The RQ values of acetamiprid, bifenthrin, etofenprox + acetamiprid and pyriproxyfen + acetamiprid were 334.6, 3.95, 38.91 and 10.33, respectively. Etofenprox + acetamiprid, pyriproxyfen + acetamiprid and bifenthrin were classified as safe (category 1) and acetamiprid was classified as slightly to moderately toxic (category 2) (Table 5).

## 4. Discussion

Mirid predators can help to manage *B. tabaci* in tomato crops [1,2]. The mirid *M. basicornis* preys on tomato pests in Brazil and may become a biological control agent for use in IPM programs [3,4,5,6,36,37]. Insecticides from different chemical groups and active ingredients for control of *B. tabaci* are commercially available. These products range from reduced-risk, which rarely harm natural enemies, to broad-spectrum, which are acutely toxic to natural enemies [38,39,40], compromising the implementation of IPM programs. To mitigate incompatibility issues, information is needed on the acute toxicity of the insecticides that are most often used to control *B. tabaci* and their effects on natural enemies.

According to our findings, buprofezin, cyantraniliprole and spiromesifen were considered reduced-risk insecticides for *M. basicornis* and classified as harmless according to the IOBC criteria (class 1), with LT_50_ values similar to the control treatment. Buprofezin is an insect growth regulator (IGR) that acts on the immature stage of sucking pests by inhibiting chitin synthesis and consequently the insects cannot molt normally [41,42]. Spiromesifen, which inhibits the acetyl CoA carboxylase, derived from tetronic and tetramic acids, interferes with the development, fecundity and lipid biosynthesis of the pest [43]. The diamide cyantraniliprole can act on nymphs and adults of sucking pests, inhibiting muscle contraction when the molecules bind to ryanodine receptors, resulting in starvation, paralysis and death [44,45]. In the present case, these insecticides did not cause acute toxicity to the natural enemy and the survival rate was also similar to the control. The results demonstrated that these insecticides are not harmful to *M. basicornis* in controlled conditions. 

Similar results were found when residues of parallel insecticides did not cause high levels of acute toxicity to *M. basicornis* adults and nymphs [18,19,20,21]. Interestingly, Wanumen et al. [18] showed that spiromesifen was innocuous to adults of *M. basicornis* exposed to residues on an inert substrate, but mortality increased in extended laboratory assays (sprayed on tomato leaves). In the present study, spiromesifen on tomato leaves retained the harmless acute effect in controlled conditions. This was elucidated by differences in the concentrations used, contributing to this negative effect. Therefore, at the semi-field level, it does not cause a lethal effect on this natural enemy [18] and probably will not be lethal in field conditions. 

Similarly to the results for *M. basicornis*, buprofezin did not have a lethal effect on adults and nymphs of *Pilophorus typicus* Distant (Hemiptera: Miridae) under controlled conditions [46] or on *Macrolophus caliginosus* Wagner (Hemiptera: Miridae) under field conditions [47]. For the predator *Deraeocoris brevis* (Uhler) (Hemiptera: Miridae), cyantraniliprole was lethal to nymphs and was less toxic to adults [48]. These studies demonstrate the importance of knowing the acute toxicity of insecticides for the integrated use of biological and chemical controls in an IPM program. The physiological effects of reduced-risk insecticides may depend on the sensitivity of a species and its life stages and, therefore, it is important to test each active ingredient on each species of natural enemy [49]. Knowledge of the sensitivity of a species in tropical conditions is important for agriculture, considering that the sensitivity can differ depending on climate, temperature and light incidence [50].

Acetamiprid induces excitation until death, acting on neurons as a competitive modulator of the nicotinic acetylcholine receptor [51,52]. It is a chlorinated neonicotinoid which mainly acts by ingestion, due to its activity inside the plants, allowing systemic translocation in the sap vessels [53]. When sprayed on tomato leaves, it caused 80% mortality in adults of another mirid predator, *Nesidiocoris tenuis* (Reuter) (Hemiptera: Miridae) after five days of exposure [54]. In addition to acting systemically due to its hydrophobicity, acetamiprid also acts by contact [55,56]. On inert substrates in controlled conditions, acetamiprid caused 100% mortality in *M. caliginosus* and *Orius laevigatus* (Fieber) (Hemiptera: Anthocoridae) [57]. Because mirid predators are omnivorous [58], systemic insecticides such as acetamiprid may affect this natural enemy by both the contact and ingestion routes of exposure. 

Etofenprox and bifenthrin affect insects mainly through contact exposure [59,60]. Etofenprox and bifenthrin modulate the sodium channel in neuron axons, which keep the insect hyperexcited and also cause death [59]. Some pyrethroids tested on piercing-sucking predators (Hemiptera) also caused acute toxicity [61,62], as did bifenthrin in this study. The active ingredient lambda-cyhalothrin is efficient in controlling the *B. tabaci* pest population in tomatoes, but significantly affected the survival of the mirid predator *N. tenuis* under laboratory conditions [61]. Deltamethrin demonstrated acute toxicity similar to bifenthrin, which caused 70% mortality in *N. tenuis* after contact with residues for more than 72 h [62]. Bifenthrin had a similar effect to our results when tested with a full concentration series bioassay in the laboratory, proving highly toxic to adults of *Geocoris punctipes* (Say) (Hemiptera: Geocoridae) and *Orius insidiosus* (Say) (Hemiptera: Anthocoridae) [63]. Pyrethroids are harmful to *Podisus nigrispinus* (Dallas) (Hemiptera: Pentatomidae), with high toxicity to nymphs and adults at the highest recommended rate in soybeans, even when mixed with low-risk insecticides [64].

Most acute toxicology studies do not address the ecological vulnerability of natural enemies to broad-spectrum pesticides and only assess whether they kill the insect, which is insufficient to recommend these pesticides in IPM programs. However, by determining the median lethal concentration (LC_50_) for the most physiologically harmful insecticides to beneficial insects, it is possible to calculate the risk quotient (RQ) to determine the ecological risks of a given insecticide to a natural enemy. The insecticides evaluated here, especially acetamiprid and bifenthrin, showed quite low LC_50_ values. Calculating the RQ values for each formulation and considering the concentration of the active ingredients, most of the RQ values were classified as safe (RQ < 50), except for acetamiprid, categorized as slightly to moderately toxic (50 < RQ ≤ 2500). These results are important to understand both the physiological and ecological risks together, in order to make decisions for IPM recommendations [65,66].

This is the first study with *M. basicornis* to assess the acute toxicity and RQ values of the insecticides that are most often used to control *B. tabaci* in tomato crops. Other studies conducted with important natural enemies in different crop systems contributed useful information IPM [26,27,35,67,68,69]. Nevertheless, the researchers also made clear that certain insecticides tested, although classified as slightly to moderately harmful, should be thoroughly evaluated for inclusion in an IPM program, as they show high acute toxicity to the predator and other species.

Insecticides can act differently in each insect species and it is therefore important to study the pesticide formulations and their effect on the natural enemies that are most frequently found and released in the crops. As an example of the action of the same insecticides on different species, in the case of parasitoids of the genus *Trichogramma*, neonicotinoids and pyrethroids were tested to determine the LC_50_ and to calculate the RQ values [27]. For *Trichogramma dendrolimi* Matsumura, *Trichogramma ostriniae* Pang et Chen and *Trichogramma chilonis* Ishii (Hymenoptera: Trichogrammatidae), the LC_50_ values for acetamiprid were 0.32, 1.37 and 0.53 g a.i. ha^−1^ and the RQ values were 188.8, 44.1 and 114.0, respectively. Therefore, acetamiprid was categorized as slightly to moderately harmful to *T. dendrolimi* and *T. chilonis* (class 2) but safe for *T. ostriniae* (class 1) [27]. In the present study, the LC_50_ were also very low for all insecticides tested, similarly to the studies with *Trichogramma* species, but the RQ values differed for acetamiprid, showing that these specific studies must be considered when assessing the insecticides’ risks to a new species of natural enemy. 

Taken together, the present results support the hypothesis that some of the insecticides tested were physiologically more harmful than others to the natural enemy. In addition, this study elucidated the ecological risks of those that proved to be physiologically harmful. Physiological and ecological risks must be considered when using IPM tools such as chemical and biological controls. If we consider only the physiological hazard, we eliminate all the other factors that can minimize the effect of these chemicals on non-target organisms in the field. These factors can potentially make pesticides more selective, based on, for example, formulation, placement, dosage and timing [64]. If the ecological risks are considered, there is a chance to match the methods to actual conditions in the tomato fields. It may be possible to use these insecticides with temporal and spatial separation [65]. IPM methods provide better results when most of the tools can be implemented in the field of the crop cycle [70].

## 5. Conclusions

The results obtained in controlled conditions for *M. basicornis* nymphs and adults are important to understand the action of insecticides on this natural enemy. Buprofezin, cyantraniliprole and spiromesifen were considered reduced-risk insecticides, but future studies should assess sublethal and transgenerational effects on this beneficial insect. Acetamiprid, bifenthrin, etofenprox + acetamiprid and pyriproxyfen + acetamiprid were harmful and considered broad-spectrum for *M. basicornis*. The physiological and ecological classifications for broad-spectrum insecticides were determined for *M. basicornis* adults and will support future IPM decisions. The RQ data provide insight into the ecological risk assessment for data acquired under more controlled conditions, but this needs to be confirmed with semi-field and field assays. Further studies are necessary to confirm compatibility of the methods with these active ingredients, such as in a greenhouse with regular insecticide spraying, to determine the persistence of the compound residues on tomato plants and the effects on the predator. It is also important to study crop management with these products to gather more accurate information.

## Figures and Tables

**Table 1:** Active ingredient, trade name, chemical group, exposure route, mode of action and field application rate of the principal insecticides used to control *Bemisia tabaci* in tomato crops in Brazil.

Active Ingredient	Trade Name	Chemical Group	Exposure Route	Mode of Action	Field Rate (g or mL 100 L^−1^)		Field Rate (g a.i. ha^−1^)
					a.i.	c.p.	
Acetamiprid	Mospilan WG	Neonicotinoid	Systemic	Competitive modulator of nicotinic acetylcholine receptors	21.8	30	87
Bifenthrin	Seizer^®^ 10 EC	Pyrethroid	Contact and ingestion	Sodium channel modulator	1.5	15	15
Buprofezin	Applaud^®^ 25 WP	Thiadiazinone	Contact	Chitin synthesis inhibitors	50	200	500
Cyantraniliprole	Benevia^®^ 10 OD	Diamide	Systemic and contact	Ryanodine receptor modulator	12.5	125	50
Etofenprox + acetamiprid	Eleitto^®^ 30 + 16.7 OD	Pyrethroid + Neonicotinoid	Systemic and contact	Sodium channel modulator + competitive modulator of nicotinic acetylcholine receptors	12 + 6.8	40	120 + 66.8
Pyriproxyfen + acetamiprid	Privilege^®^ 10 + 20 OD	Pyridyloxypropyl ether + Neonicotinoid	Contact, ingestion, translaminar and systemic	Juvenile hormone mimics + Nicotinic acetylcholine receptor (NACHR) competitive modulators	3 + 6	30	30 + 60
Spiromesifen	Oberon^®^ 24 SC	Cetoenol	Contact and ingestion	Inhibitors of acetyl CoA carboxylase	14.4	60	144

EC (Emulsifiable Concentrate); OD (Oil Dispersion); SC (Suspension Concentrate); WG (Water-dispersible Granules); WP (Wettable Powder); c.p. (commercial product); a.i. (active ingredient).

**Table 2:** Number of live third-instar nymphs (mean ± SE) of *Macrolophus basicornis* (24, 48 and 72 h) after contact with insecticide residues on tomato leaves, corrected mortality (M_a_) after 72 h and IOBC classification of insecticides.

Treatment	Number of Live Nymphs (*n* = 15)			M_a_ (%) *	Class ^1^
	24 h	48 h	72 h		
Control	14.5 ± 0.3 a	14.0 ± 0.3 a	13.8 ± 0.3 a	-	-
Acetamiprid	3.3 ± 1.7 b	1.0 ± 0.7 b	0.2 ± 0.2 b	99.9	4
Bifenthrin	4.2 ± 1.3 b	0.7 ± 0.5 b	0.0 ± 0.0 b	100.0	4
Buprofezin	14.5 ± 0.2 a	14.0 ± 0.2 a	13.8 ± 0.3 a	0.0	1
Cyantraniliprole	14.5 ± 0.2 a	14.0 ± 0.5 a	13.6 ± 0.5 a	0.8	1
Etofenprox + acetamiprid	4.0 ± 0.9 b	0.7 ± 0.3 b	0.2 ± 0.2 b	99.9	4
Pyriproxyfen + acetamiprid	3.0 ± 1.1 b	1.7 ± 0.9 b	1.2 ± 0.9 b	87.4	4
Spiromesifen	14.8 ± 0.2 a	14.6 ± 0.2 a	14.0 ± 0.4 a	0.0	1
χ^2^	37.3	37.6	39.0	-	-
*df*	7	7	7	-	-
*p*	<0.001	<0.001	<0.001	-	-

Means followed by the same letter in a column do not differ by the Bonferroni test. * Corrected mortality (M_a_) by the Schneider–Orelli formula [34]. ^1^ Toxicological class according to IOBC (“International Organization for Biological and Integrated Control of Noxious Animals and Plants, West Palearctic Regional Section”) in which: class 1 = harmless (M_a_ < 25%); class 2 = slightly harmful (25 ≤ M_a_ ≤ 50%); class 3 = moderately harmful (51 ≤ M_a_ ≤ 75%); and class 4 = harmful (M_a_ > 75%) [25].

**Table 3:** Number of live adults (mean ± SE) of *Macrolophus basicornis* (24, 48 and 72 h) after contact with insecticide residues on tomato leaves, corrected mortality (M_a_) after 72 h and IOBC classification of insecticides.

Treatment	Number of Live Adults (*n* = 15)			M_a_ (%) *	Class ^1^
	24 h	48 h	72 h		
Control	14.5 ± 0.2 a	14.5 ± 0.2 a	14.3 ± 0.3 a	-	-
Acetamiprid	4.1 ± 1.1 c	2.2 ± 0.9 c	0.2 ± 0.2 c	98.6	4
Bifenthrin	6.8 ± 0.8 b	5.2 ± 0.8 b	2.8 ± 0.3 b	80.3	4
Buprofezin	14.0 ± 0.4 a	14.0 ± 0.4 a	14.0 ± 0.4 a	2.1	1
Cyantraniliprole	14.3 ± 0.3 a	14.0 ± 0.4 a	13.8 ± 0.5 a	3.5	1
Etofenprox + acetamiprid	6.2 ± 0.9 b	2.6 ± 1.0 c	0.5 ± 0.3 c	96.5	4
Pyriproxyfen + acetamiprid	7.8 ± 0.9 b	5.2 ± 0.9 b	1.8 ± 0.5 b	87.4	4
Spiromesifen	14.5 ± 0.2 a	14.3 ± 0.2 a	14.3 ± 0.2 a	0.0	1
CV (%)	17.9	19.8	12.9	-	-
*F*	34.9	60.9	288.2	-	-
*df*	7	7	7	-	-
*p*	<0.001	<0.001	<0.001	-	-

Means followed by the same letter in a column do not differ by the Scott–Knott test. * Corrected mortality (M_a_) by the Schneider-Orelli formula [34]. ^1^ Toxicological class according to IOBC (“International Organization for Biological and Integrated Control of Noxious Animals and Plants, West Palearctic Regional Section”) in which: class 1 = harmless (M_a_ < 25%); class 2 = slightly harmful (25 ≤ M_a_ ≤ 50%); class 3 = moderately harmful (51 ≤ M_a_ ≤ 75%); and class 4 = harmful (M_a_ > 75%) [25].

**Table 4:** Median lethal time (LT_50_) in days for third-instar nymphs and adults of *Macrolophus basicornis* after 72 h in contact with insecticide residues on tomato leaves.

Treatment	LT_50_ (95% CI)			
	Third-Instar Nymphs		Adults	
Control	55.0 (48.8–61.1)	a	26.4 (23.7–29.1)	a
Acetamiprid	1.9 (1.5–2.2)	b	2.2 (1.7–2.5)	b
Bifenthrin	2.1 (1.7–2.5)	b	3.2 (2.6–3.8)	b
Buprofezin	51.1 (45.1–57.1)	a	28.5 (25.1–31.8)	a
Cyantraniliprole	58.9 (52.7–65.2)	a	26.5 (23.3–29.8)	a
Etofenprox + acetamiprid	2.1 (1.7–2.4)	b	2.5 (2.1–2.9)	b
Pyriproxyfen + acetamiprid	1.8 (1.5–2.1)	b	3.2 (2.8–3.6)	b
Spiromesifen	55.4 (49.5–61.3)	a	22.6 (20.5–24.7)	a
χ^2^	686.96		661.1	
*df*	7		7	
*p*	<0.001		<0.001	

Means followed by the same letter in a column do not differ by the Holm-Sidak test. Cl: Confidence interval with 95% probability.

**Table 5:** Median lethal concentration (LC_50_) of insecticides for adults of *Macrolophus basicornis* after contact with residues on tomato leaves for 72 h.

Insecticides	LC_50_ (95% CI) (mg a.i. L^−1^)	χ^2^	*df*	RQ	Category *
Acetamiprid	0.26 (0.16–0.35)	11.58	4	334.6	2
Bifenthrin	0.38 (0.29–0.48)	30.34	7	3.95	1
Etofenprox + acetamiprid	4.80 (3.28–6.31)	32.07	5	38.91	1
Pyriproxyfen + acetamiprid	8.71 (6.18–11.25)	65.86	4	10.33	1

Data observed and predicted by the binomial model test with log-logistic regression. *p* < 0.0001. Cl: confidence interval with 95% probability. * Risk quotient categories according to the values at which the insecticides were classified as safe (RQ < 50), slightly to moderately toxic (50 < RQ ≤ 2500), or dangerously toxic (RQ > 2500) [26].

## Data Availability

The data presented in this study are available in the Supplementary Materials section.

## Supplementary Materials

The following are available online at https://www.mdpi.com/article/10.3390/insects12121092/s1, Figure S1: Detail of experimental units with flasks used to insert the petiole of each tomato leaf to maintain turgidity during the bioassay and the cage covered with voile fabric.
