# Supplementary material for: Risk Assessment of Insecticides Used in Tomato to Control Whitefly on the Predator Macrolophus basicornis (Hemiptera: Miridae)
Source: Insects. 2021 Dec 7;12(12):1092. doi: 10.3390/insects12121092 (PMC8709244; doi:10.3390/insects12121092)
Supplement: Supplementary file 1 [file insects-12-01092-s001.zip › insects-1471532-supplementary.pdf]

Risk assessment of insecticides used in tomato to control whitefly on the predator *Macrolophus basicornis* (Hemiptera: Miridae)

Supplementary Material

Include:
Figure S1: Detail of experimental units with flasks used to insert the petiole of each tomato leave to maintain turgidity during the bioassay and the cage covered with voile fabric.

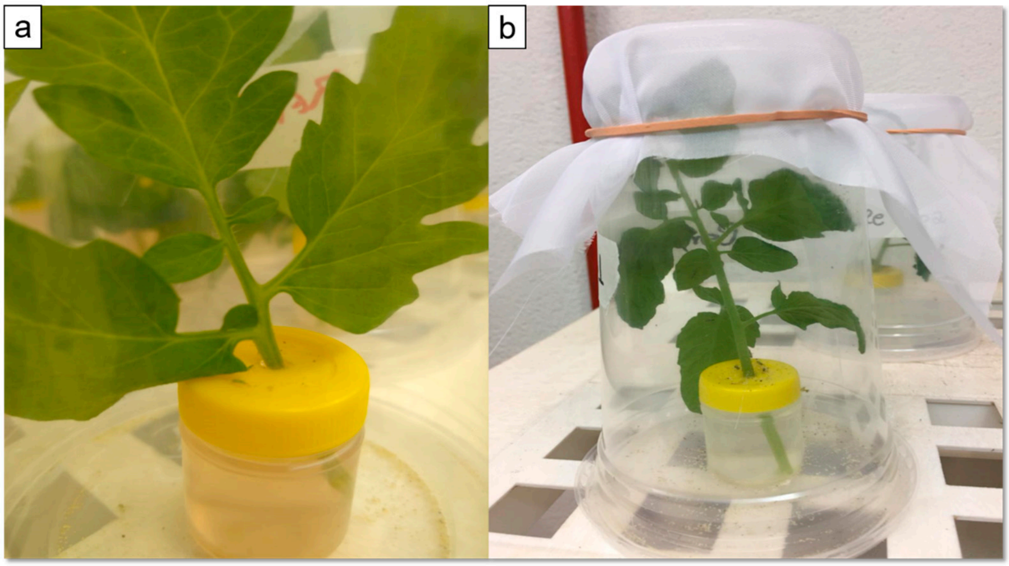

Figure S1 a) Detail of the tomato leave inserted in the orifice of the flask (20 mL) with a lid containing an orifice, previously filled with water to maintain turgidity during the bioassay; b) the flask with the leave inside the cage (12 cm high × 5 cm diameter) covered with voile fabric to prevent accumulation of toxic gases and retain the insects, representing the experimental unit.
